# Hepatic Transcriptomics Reveals that Lipogenesis Is a Key Signaling Pathway in Isocitrate Dehydrogenase 2 Deficient Mice

**DOI:** 10.3390/genes10090728

**Published:** 2019-09-19

**Authors:** Jeong Hoon Pan, Jingsi Tang, Mersady C. Redding, Kaleigh E. Beane, Cara L. Conner, Yun Jeong Cho, Jiangchao Zhao, Jun Ho Kim, Byungwhi C. Kong, Jin Hyup Lee, Jae Kyeom Kim

**Affiliations:** 1 School of Human Environmental Sciences, University of Arkansas, Fayetteville, AR 72701, USA; jhpan@udel.edu (J.H.P.); jt031@uark.edu (J.T.); mcreddin@uark.edu (M.C.R.); kebeane@uark.edu (K.E.B.); clc043@uark.edu (C.L.C.); yjcho@uark.edu (Y.J.C.); 2 Department of Animal Science, University of Arkansas, Fayetteville, AR 72701, USA; jzhao77@uark.edu; 3 Department of Food Science and Biotechnology, Andong National University, Andong 36729, Korea; jhkim@anu.ac.kr; 4 Poultry Science, University of Arkansas, Fayetteville, AR 72701, USA; 5 Department of Food and Biotechnology, Korea University, Sejong 30019, Korea

**Keywords:** Idh2, hepatic transcriptomics, lipid metabolism, bioinformatics, knockout mouse

## Abstract

Mitochondrial nicotinamide adenine dinucleotide phosphate (NADP^+^)-dependent isocitrate dehydrogenase (IDH2) plays a key role in the intermediary metabolism and energy production via catalysing oxidative decarboxylation of isocitrate to α-ketoglutarate in the tricarboxylic acid (TCA) cycle. Despite studies reporting potential interlinks between IDH2 and various diseases, there is lack of effort to comprehensively characterize signature(s) of IDH2 knockout (IDH2 KO) mice. A total of 6583 transcripts were identified from both wild-type (WT) and IDH2 KO mice liver tissues. Afterwards, 167 differentially expressed genes in the IDH2 KO group were short-listed compared to the WT group based on our criteria. The online bioinformatic analyses indicated that lipid metabolism is the most significantly influenced metabolic process in IDH2 KO mice. Moreover, the TR/RXR activation pathway was predicted as the top canonical pathway significantly affected by IDH2 KO. The key transcripts found in the bioinformatic analyses were validated by qPCR analysis, corresponding to the transcriptomics results. Further, an additional qPCR analysis confirmed that IDH2 KO caused a decrease in hepatic de novo lipogenesis via the activation of the fatty acid β-oxidation process. Our unbiased transcriptomics approach and validation experiments suggested that IDH2 might play a key role in homeostasis of lipid metabolism.

## 1. Introduction

Isocitrate dehydrogenases (IDHs) catalyze the oxidative decarboxylation of isocitrate into α-ketoglutarate [1] and are classified into two subclasses based on a type of cofactors utilized in the reaction. Either nicotinamide adenine dinucleotide (NAD^+^) or NADP^+^ is utilized as a cofactor to form NADH (reduced form of NAD^+^) or NADPH (reduced form of NADP^+^), respectively [2]. There are three isoforms of IDHs depending upon their localization: Cytosolic NADP^+^-dependent isocitrate dehydrogenase (IDH1), mitochondrial NADP^+^-dependent isocitrate dehydrogenase (IDH2), and mitochondrial NAD^+^-dependent isocitrate dehydrogenase (IDH3) [3]. Of the three isoforms, IDH1 and IDH2 produce NADPH, which is a molecule required not only for the regeneration of glutathione [4], but also for the utilization of anabolic pathways, such as fatty acid elongation processes [5]. Regarding NADPH production, IDH2 plays a critical role in mitochondrial homeostasis since glucose 6-phosphate dehydrogenase, another key enzyme participating the pentose phosphate pathway to produce NADPH, is lacking in mitochondria [1].

Thus far, a few studies have utilized IDH2 deficient in vitro or in vivo models. In non-cancer studies, the mechanistic contributions of IDH2 are fairly clear as to ROS homeostasis which might be related to NADPH production and glutathione regeneration. Multiple studies have shown that IDH2 knockout (KO) mice elicited enhanced disease susceptibility ulcerative colitis [6], cardiac hypertrophy [7], and others [8,9,10]. Most these studies explained the mechanisms by which IDH2 deficiency worsens disease susceptibility through the imbalances of the mitochondrial redox status [1,11]. In agreement with these studies, in a recent publication, the inactivation of IDH2 or deletion of *Idh2* increased cytosolic reactive oxygen species (ROS), thereby potentiating oxidative damages to DNA, proteins, and lipids in ischemia induced liver damages [9]. Of note, the enzymatic activity of IDH2 can be affected by various factors, such as chemicals [12], diet [13] in addition to post-translational regulation [14]. Despite important physiological roles of IDH2 in energy metabolism in the TCA cycle, most studies however are overly weighted towards redox systems and are thus somewhat biased. To this end, the global gene expression analyses, such as mRNA sequencing (RNAseq), allows researchers to unravel novel function(s) and identify candidate genes associated with characteristics of interest, in our case, IDH2 deficiency.

To the best of the authors’ knowledge, a global gene expression analysis in IDH2 KO mice has never been undertaken, in spite of studies reporting potential interlinks between this gene and various diseases as aforementioned. Therefore, the present study was conducted to capture metabolic signatures of IDH2 KO liver transcriptome using RNAseq which may provide information to discover new transcripts and to quantify transcript levels [15]. Further, using the RNAseq dataset, a gene ontology (GO) analysis and software-based pathway analysis were performed to further identify key biological functions of IDH2 influencing disease-related signaling pathways.

## 2. Materials and Methods

### 2.1. Animal Housing, and Study Design

**Figure 1:**
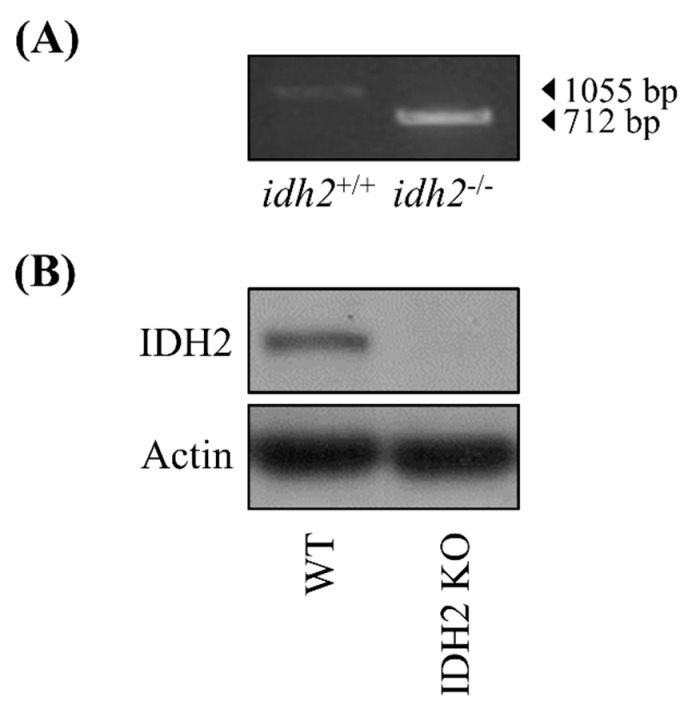
The validation of *Idh2* deletion in mice. The ablation of *Idh2* was verified by tail DNA genotyping method (**A**), and by comparing IDH2 protein expression in liver tissues of wild-type (WT) and IDH2 KO mice (**B**).

The animals used in this study were idh2^−/−^ germ-line knockout (IDH2 KO) mice (both male (*n* = 2) and female (*n* = 3)) and their littermate wild type (WT) mice (both male (*n* = 3) and female (*n* = 4)). Both WT and IDH2 KO mice share same C57BL/6N stain background. All mice were maintained in cages (up to 4 mice per cage) in a windowless room with a 12 h-light–dark cycle at a constant temperature of 23 ± 2 °C and humidity of 40 ± 10%. Mouse tail genotyping was carried out before use (Figure 1A) and further confirmed by measuring the IDH2 protein expression (Figure 1B). All mice were killed by exsanguination at 8–10 weeks of age via cardiac puncture under anesthesia using 2,2,2-tribromoethanon (Sigma-Aldrich, St. Louis, MO, USA). The harvested liver tissues were quickly placed in the RNALater solution (Invitrogen, Carlsbad, CA, USA) for 12 h at 4 °C, and then were stored at −80 °C until further RNA isolation. All animal handling and experiments were performed in accordance with a protocol approved by the Institutional Animal Care and Use Committee of the University of Arkansas (IACUC protocol approval number: 17044).

### 2.2. Liver Transcriptomics Analysis

The total RNA from liver tissues was isolated using the RNeasy Plus Mini Kit (Qiagen, Hilden, Germany) and then the quality of isolated RNA was first assessed using the conventional A260/280 ratio and A260/230 ratio measurement (SpectraMax i3x; Molecular Devices, Sunnyvale, CA, USA) which were all satisfactory. In addition, the RNA Integrity Number (also known as RIN) was assessed using the RNA R6K assay for the Agilent 2200 TapeStation (Agilent Technology, Santa Clara, CA, USA) in which RINs ranged between 8.5 and 9.5 and were hence satisfactory. For the RNAseq analysis, library preparation and sequencing analyses were carried out at the Research Technology Support Facility of Michigan State University (East Lansing, MI, USA). Liver transcriptome (*n* = 7 for WT group and *n* = 5 for IDH2 KO group) was analyzed using a 1 × 50 bp single end read method of Illumina HiSeq system (Illumina Inc., San Diego, CA, USA) as described in an earlier report [16]. The total read counts were normalized by the transformation to log_2_ number of reads per million in order to stabilize the variance. The normalized reads per million values were then subjected to further statistical analyses followed by computational bioinformatics. The sequencing dataset supporting this article has uploaded to the Gene Expression Omnibus repository (GEO; https://www.ncbi.nlm.nih.gov/geo/query/acc.cgi?acc=GSE128361), which will be published on 15 March 2020.

### 2.3. Bioinformatics Analyses

**Figure 2:**
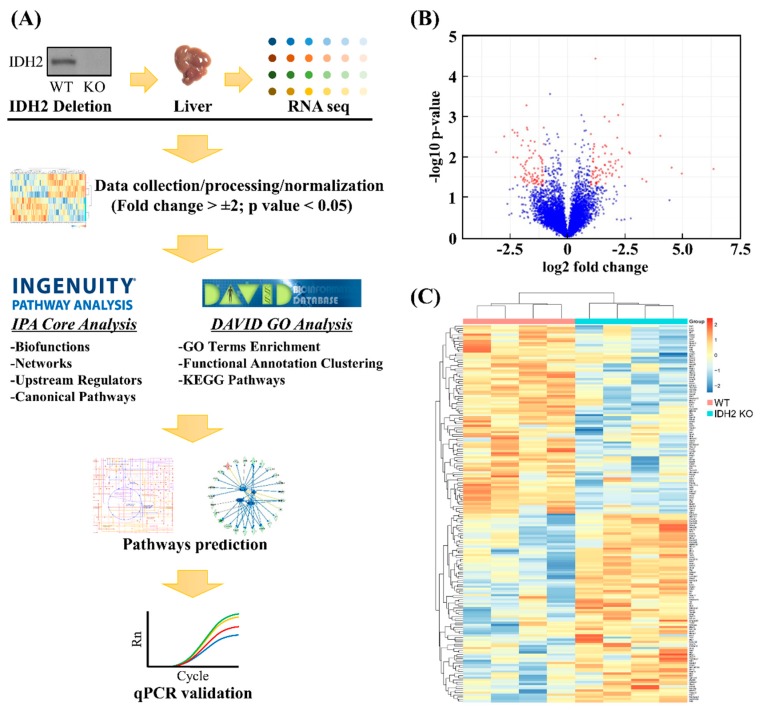
Procedures of hepatic transcriptomics dataset. The analyses of transcriptomics dataset followed by computational analyses and qPCR validation: A flowchart (**A**), A volcano plot (**B**), and heat map (**C**) showed differentially expressed genes and the relationships among samples used, respectively.

For the transcriptomics dataset, a short list of differentially expressed genes (DEGs) was produced based on the criteria: mRNAs showing *p*-value < 0.05 and fold change > 2. To visualize fold changes in the transcriptome dataset in response to *Idh2* deletion compared to the WT mice, a volcano plot and heat map were generated using the R package software (R Studio 3.5.2 version; The R Foundation, Boston, MA; available at r-project.org). The heat map was generated for the short-listed mRNAs by creating a normalized z-score that was calculated in the R. The short-listed DEGs were subjected to the DAVID, which is web-based tools to identify enriched biological themes including GO terms, group functionally related genes, cluster annotation terms for large gene lists and pathways in the KEGG database. The functional annotation clustering algorithm was used to generate a clustered, non-redundant report of related annotation terms, and the groups of annotation clusters with gene count threshold > 10, and EASE scores (i.e., modified Fisher Exact *p*-value for gene-enrichment analysis) < 0.01 were retained. Similarly, the short list was subjected to the IPA software (Qiagen) to perform a core analysis in order to identify the upstream regulators for DEGs and related canonical pathways in response to *Idh2* deletion. The IPA core analyses are based on previous knowledge of the associations of upstream regulators and their downstream target genes archived in the Ingenuity Knowledge Base. The *p*-values were calculated by Fisher’s exact test for the upstream regulator analysis. A schematic diagram of the data acquisition, bioinformatics analyses, and validation experiments is shown in Figure 2A.

### 2.4. Quantitative RT-PCR Validation

The transcriptomics and computational bioinformatics analyses were validated by quantitative RT-PCR analysis using StepOnePlus system (Applied Biosystems; Foster City, CA, USA). Two μg of total RNA was reverse transcribed using the High Capacity cDNA Reverse Transcription Kit (Applied Biosystems) according to the manufacturer’s protocol. The reaction mixture contained TaqMan Gene Expression Mastermix, respective primer(s) tagged with TaqMan probes (Table S5), and cDNA. The amplification was conducted under the following conditions: One cycle at 50 °C for 2 min and 95 °C for 10 min, followed by 40 cycles of denaturation (95 °C for 15 s) and annealing/extension (60 °C for 1 min). The genes of interest were normalized to that of the reference gene (*Actb*). The data were analyzed with StepOne Software (Ver. 2.1; Applied Biosystems) using the 2^−ΔΔCT^ method.

### 2.5. Statistical Analyses

All data were analyzed by a two tailed, Welch’s t test. A *p*-value of 0.05 or less was considered statistically significant (SAS 9.4 version; SAS Institute Inc., Cary, NC, USA).

## 3. Results

### 3.1. Transcriptome Profiles and Identification of DEGs

A total of 12 RNAseq libraries were constructed using RNA samples of liver tissues from both wild-type (WT) and IDH2 KO mice. After filtering low read counts and normalization, a total of 6583 transcripts remained. Of the 6583 genes, only mRNAs showing *p*-value < 0.05 and fold change >2 were considered differentially expressed genes (DEGs) for further bioinformatics analyses and qPCR validation (Figure 2A). Using these criteria, this study was able to select 167 DEGs as shown in a volcano plot (Figure 2B). The red dots indicate that the DEGs passed the criteria (i.e., *p*-value < 0.05, and fold change >2). The complete gene list is provided in the Table S1. Further, the gene expression profiles of DEGs were visualized using a heat map (Figure 2C). A cluster dendrogram for two biological replicates (i.e., WT and IDH2 KO mice) shows clear segregation between the 77 upregulated (red keys) and 90 downregulated genes (blue keys).

### 3.2. Computational Bioinformatic Pathway Prediction of DEGs

**Figure 3:**
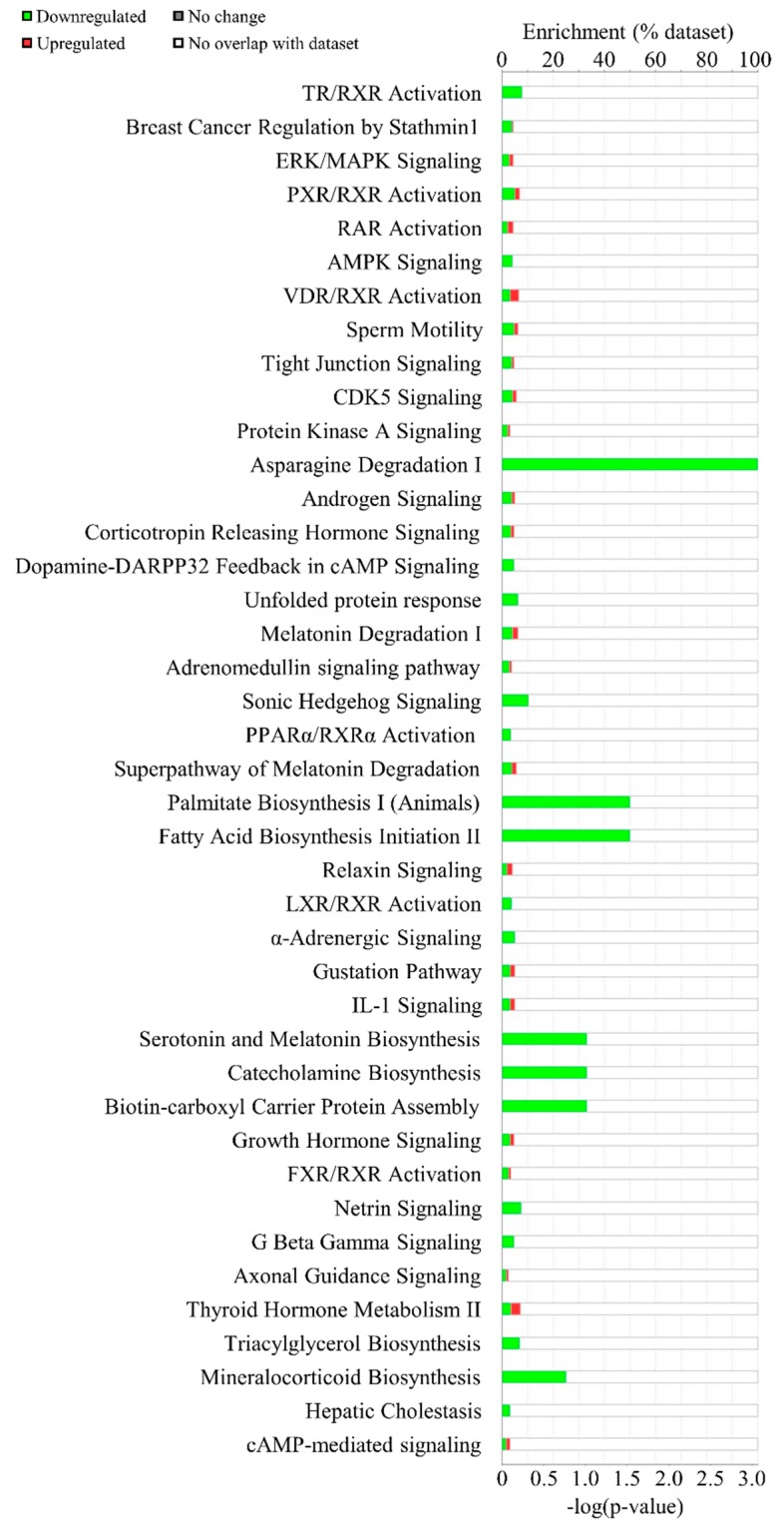
Canonical pathway analysis using Ingenuity Pathway Analysis software. Enriched canonical pathways in IDH2 KO mice liver were listed. The green bars indicate the number of downregulated genes while the red portion in bars show the number of upregulated genes.

To explore the key pathways in which 167 DEGs were involved and enriched, the computational bioinformatic pathway prediction analyses were performed using core analysis function of both Kyoto Encyclopedia of Genes and Genomes (KEGG) and Ingenuity Pathway Analysis (IPA) software (Qiagen). In the core analysis, the canonical pathway analysis function ranked the most enriched pathways of the DEGs based on *p*-values and the number of overlapping genes within the list of DEGs. In this, the top 5 canonical pathways enriched in DEGs include TR/RXR activation (*p* = 0.0003), breast cancer regulation by Stathmin1 (*p* = 0.0040), ERK/MAPK signaling (*p* = 0.0044), PXR/RXR activation (*p* = 0.0046), and RAR activation (*p* = 0.0049) (Figure 3).

**Figure 4:**
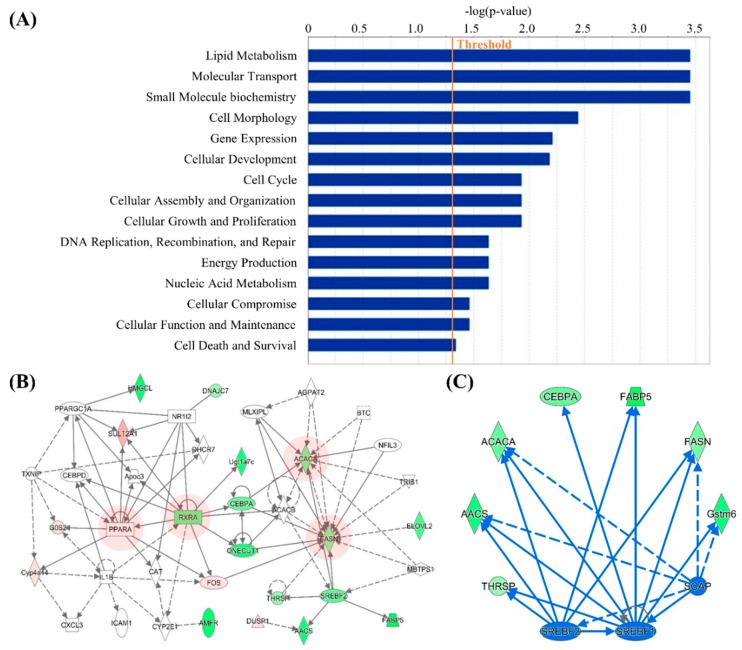
Biofunctions, networks, and upstream analyses using the Ingenuity Pathway Analysis (IPA). Enriched functional roles of networks in IDH2 KO mice are listed (**A**), and IPA Networks only related to lipid metabolism are integrated. The key transcripts at core position of the networks are highlighted with red color (**B**). Upstream regulators including *Srebf1*, *Srebf2*, and *Scap*, were predicted in the interactome image (**C**).

In order to obtain further insights into the interaction networks related with IDH2 KO, the potential functional networks of DEGs were predicted. The IPA network analysis revealed that the DEG can be clustered into 9 significant networks (Table 1). Each network was automatically scored and ranked by the IPA software based on the number of network eligible molecules. The primary functional roles (labeled as Top Biofunctions in the Table 1) of top three networks include lipid metabolism, molecular transport, and small molecule biochemistry (Figure 4A). Of the 9 networks, due to their similar Top Biofunctions, the top two networks were integrated and visualized in the Figure 4B. As shown, the key genes in lipogenesis (i.e., *Ppara*, *Rara*, *Acaca*, and *Fasn*) were found in the integrated network, and these genes are at the core position of the network as they are associated with neighboring factors.

Subsequently, to identify potential regulatory upstream gene(s), the upstream analysis of the IPA was utilized. In this, three upstream regulators *Srebf1*, *Srebf2*, and *Scap* (Activation z-scores: −2.527, −2.200, and −2.207, respectively) were able to be found, which were suppressed in live tissues of IDH2 KO mice. Three common target molecules in the DEGs, namely *Aacs*, *Acaca*, and *Fasn*, were regulated by all three upstream regulators (Figure 4C).

### 3.3. Database for Annotation, Visualization, and Integrated Discovery (DAVID) Web-Based Bioinformatic Analysis of DEGs

**Figure 5:**
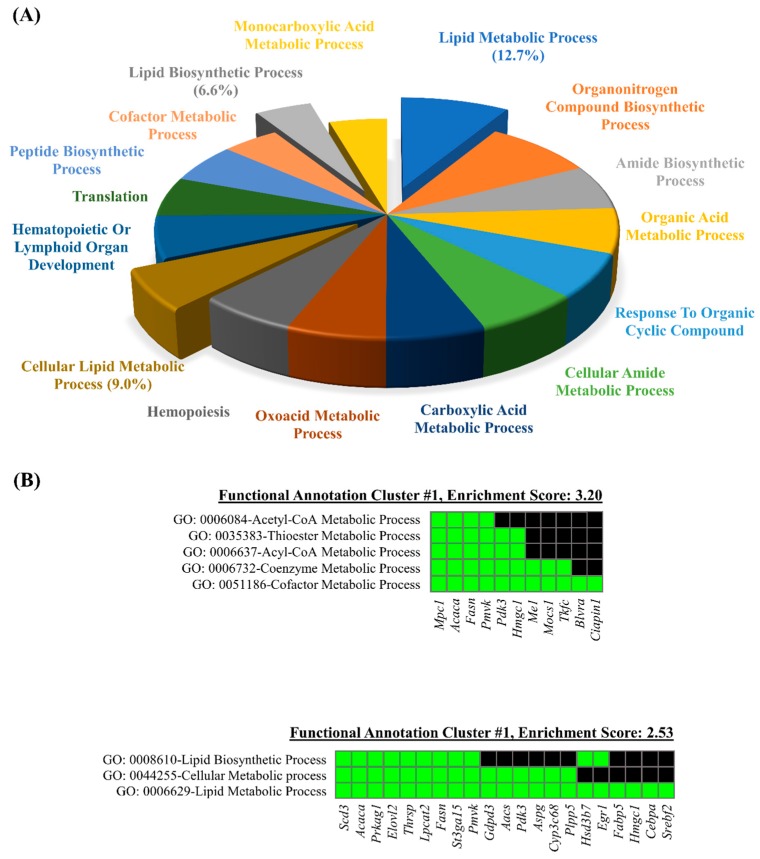
Functional analyses of differentially expressed genes by DAVID gene ontology (GO) enrichment analysis. The representative GO terms for biological processes (**A**) were utilized for DAVID functional annotation clustering of categories (**B**).

The DAVID GO terms enrichment analysis was carried out using the list of DEGs. The results demonstrated that 132 genes (79.5%, 132/167) were significantly enriched in the biological process category. A total of 139 GO terms for the biological category were annotated, and 16 GO terms were retrieved with the gene counts >10 and EASE Score < 0.01. The list of 16 GO terms with respective *p*-values is provided in the Table S2. As shown in the Table S2, the lipid metabolic process (GO: 0051186) showed the highest significant enrichment (by enrichment 12.7%), followed by organonitrogen compound biosynthesis process (GO: 1901566) and amide biosynthetic process (GO: 0043604). A pie chart depicts contributions of enriched biological processes and three processes related to lipid metabolism were highlighted which account for 28.3% of total (Figure 5A).

Additionally, the DAVID functional annotation clustering of the GO terms in the biological process category was performed. The lists of particular genes for the most enriched clusters are shown (Figure 5B), and the annotation clusters are listed in Table S3. The annotation clustering analysis revealed that the Acyl-CoA metabolic process (*p* = 0.00032), the Thioester metabolic process (*p* = 0.00032), the cofactor metabolic process (*p* = 0.00042), the Acetyl-CoA metabolic process (*p* = 0.00120), and the coenzyme metabolic process (*p* = 0.00190) were the most enriched annotation clusters, followed by the annotation cluster 2 which includes the lipid metabolic process (*p* = 0.00053), the lipid biosynthetic process (*p* = 0.00680), and the cellular lipid metabolic process (*p* = 0.00710) (Figure 5B). Taken together, the lipid metabolic process was the common enriched term in both the DAVID GO terms enrichment analysis and the functional annotation clustering analysis.

**Figure 6:**
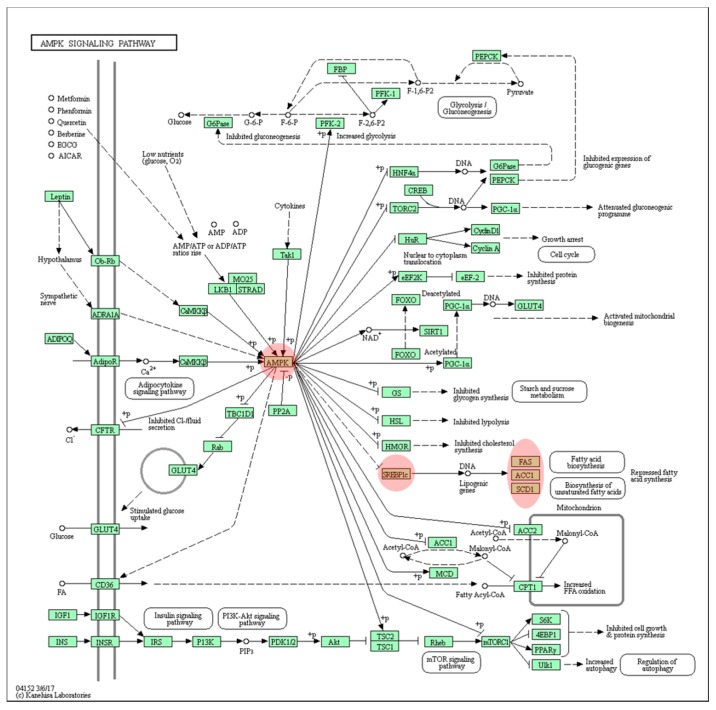
KEGG AMPK signaling. The AMPK signaling pathway was predicted as the most enriched pathway from the KEGG pathways analysis provided by the DAVID tools. The key player genes are highlighted with red color.

Last, the DEGs were subjected to the KEGG pathway analysis, which uses the most diverse tools among the multiple pathway databases. In the KEGG pathway database, the AMPK signaling pathway (*p* = 0.005) was found to be the most enriched (shown in the Figure 6) followed by fatty acid metabolism (*p* = 0.010), the PPAR signaling pathway (*p* = 0.034), and the insulin signaling pathway (*p* = 0.035) (Table S4).

### 3.4. Quantitative PCR Validation of Predicted Pathway of DEGs

**Figure 7:**
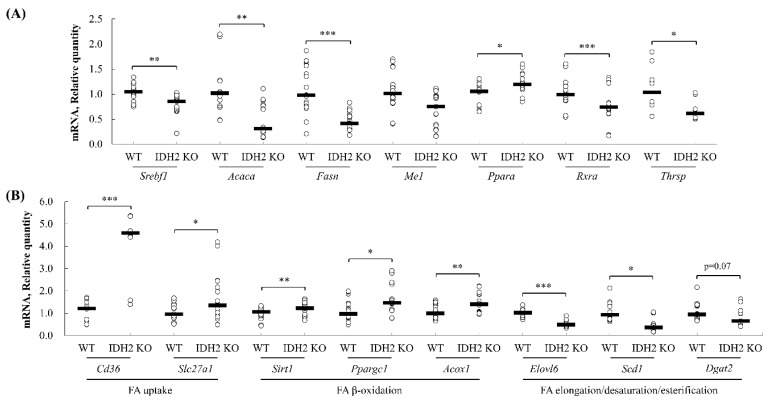
The validation of RNA sequencing results using quantitative PCR. The genes found at the core positions of the networks, upstream regulators, canonical pathway (TR/RXR), and found in the functional annotation clustering of DAVID tools were randomly selected and validated using qPCR analyses. The data were expressed as the means ± standard error of means (SEM; *n* = 5 for male, and *n* = 7 for female) (**A**). In addition to the validation, the representative genes of each lipid metabolic process were randomly selected and their expression levels were assessed. The genes responsible for fatty acid uptake (*Cd36*, and *Slc27a1*), fatty acid β-oxidation (*Sirt1*, *Ppargc1*, and *Acox1*), fatty acid esterification (*Dgat2*), and fatty acid desaturation/elongation (*Scd1*, and *Elovl6*) were quantified using qPCR analysis (**B**). * *p* < 0.05; ** *p* < 0.01; *** *p* < 0.001.

The genes found from (1) core positions in the integrated networks (retrieved from the upstream analysis, and canonical pathway analysis of IPA) and (2) the functional annotation clustering analysis of the DAVID tools were selected and validated using qPCR analysis to assess the reliability of RNAseq results and computational predictions. First, the core positioned genes in the gene networks (Figure 4B), *Rxra*, *Acaca*, *Fasn*, and *Ppara*, were quantified by qPCR analysis. The expression levels of *Rxra*, *Acaca*, and *Fasn* were significantly downregulated in IDH2 KO mice liver, but *Ppara* expression was significantly upregulated. These expression patterns are all in agreement with our RNAseq dataset (Figure 7A). Additionally, an upstream regulator, responsible for the de novo lipogenesis pathway, *Srebf1*, was also significantly downregulated by 16.7% in IDH2 KO mice liver (Figure 7A) as predicted by the IPA. Furthermore, *Me1* and *Thrsp* were validated, as these two genes were both shown in both the IPA and DAVID analyses and are included in our list of DEGs. The expression of both *Me1* and *Thrsp* was significantly downregulated in IDH2 KO mice liver thus consistent with the RNAseq dataset (Figure 7A). In order to further validate computational predictions, additional representative genes related to lipid metabolism were measured. As a result, the genes responsible for the fatty acid uptake (i.e., *Cd36*, and *Slc27a1*) and fatty acid β-oxidation (i.e., *Sirt1*, *Ppargc1*, and *Acox1*) were all significantly upregulated (Figure 7B), whereas the genes responsible for fatty acid elongation (i.e., *Elovl6*) and fatty acid desaturation (i.e., *Scd1*) were all downregulated in IDH2 KO mice liver tissues (Figure 7B). A fatty acid esterification gene (i.e., *Dgat2*) showed marginal difference (*p* = 0.07). Overall, our qPCR results showed consistent expression patterns to RNAseq results and computational bioinformatic predictions.

## 4. Discussion

Multiple studies have demonstrated the implications of IDH2 in diverse disease models, such as colitis and non-alcoholic liver disease [6,10,17]. However, most studies suggested that increased disease susceptibility might be due to a compromised mitochondrial redox status in IDH2 KO mice. Thus far, however, no study has attempted to comprehensively characterize metabolic signature(s) of IDH2 KO mice. To this end, this study analyzed the hepatic transcriptome profiles of the IDH2 KO mice followed by computational pathway analyses in order to explore key functional genes and pathways that are significantly impacted when *Idh2* is deficient. As a result, the transcriptome analysis showed that overall genetic signatures were substantially altered in IDH2 KO mice livers. More specifically, the computational analytic tools, including DAVID, KEGG, and IPA software used in this study all suggested that lipid metabolism is a significantly altered signature of IDH2 KO liver transcriptome.

In our IPA canonical pathway analysis, the TR/RXR activation pathway was found to be most significantly enriched in IDH2 KO mice (Figure 3). The downstream genes of the TR/RXR activation pathway included *Fasn*, and *Acaca* that are both related to lipogenesis and decreased expressions of the genes were confirmed in our qPCR validation (*p* < 0.01 and *p* < 0.001, respectively; Figure 7A). These results are consistent with the IPA predictions. The thyroid hormone itself is one of the master regulators of de novo lipogenesis [18]. The thyroid hormone acts through thyroid hormone receptors which bind to a thyroid hormone response elements as a form of heterodimer with a retinoid X receptor (or RXR), thereby orchestrating gene expressions related to fat metabolism [19]. In the absence of the thyroid hormone, TR coupled with a corepressor binds to the hormone response elements to negatively regulate gene expression [20]. In the present study, lipogenesis genes of the TR/RXR pathway were significantly downregulated in IDH2 KO mice liver tissues, suggesting that *Idh2* might affect lipogenesis via modulating the canonical TR/RXR pathway.

In order to better understand potential cross-talks between signaling networks, the network analysis of the IPA core analysis was also carried out. In this, the top two networks both included the lipid metabolism biofunction (Table 1). To visualize the networks, this study highlighted lipid metabolism biofunction genes in Figure 4B that include *Acaca*, *Fasn*, and *Elovl.* The de novo lipogenesis is a metabolic pathway of fatty acid synthesis from excessive energy intake (e.g., carbohydrates) and multiple regulatory genes are involved in this pathway. For example, Acaca (also known as acetyl-CoA carboxylase-1; encoded by the *Acaca*) is a key enzyme of de novo lipogenesis [21]. Acetyl-CoA is a substrate of Acaca to synthesize malonyl-CoA which is an essential intermediate metabolite for fatty acid synthesis. The liver specific *Acaca* deletion significantly reduced lipid accumulation, emphasizing the importance of hepatic *Acaca* in de novo lipogenesis [22]. Subsequently, *Fasn* also plays a key role by converting the acetyl-CoA and malonyl-CoA into palmitate, the end product of glucose metabolism [23]. This end product can be esterified to produce triacylglycerol for further storage [23].

Further, the qPCR assays for additional representative genes of lipid metabolism showed that the expression patterns of genes responsible for fatty acid uptake (*Cd36*, and *Slc27a1*), lipogenesis (*Srebf1*, *Acaca*, and *Fasn*), fatty acid esterification (*Dgat2*), fatty acid β-oxidation (*Sirt1*, *Ppargc1*, and *Acox1*), and fatty acid desaturation/elongation (*Scd1*, and *Elovl6*) were in good agreement with current transcriptomics results (Figure 7A,B). In line with this, our previous report also showed a trend of a decrease in hepatic lipogenesis mRNAs (i.e., *Srebf1*, *Fasn*, and *Dgat2*) in female IDH2 KO mice [17]. Additionally, it was reported that IDH2 deficient mice are resistant to obesity and hepatic steatosis via the suppression of lipogenesis and the elevation of thermogenesis in the liver and adipose tissues [8,24]. These findings all support our results that IDH2 KO might suppress lipogenesis in the liver.

The upstream analysis revealed that the three upstream regulators including *Srebf1*, *Srebf2*, and *Scap* (activation z-scores: −2.527, −2.200, and −2.207, respectively) were suppressed in IDH2 KO mice livers (Figure 4C). Importantly, there were three common molecules (i.e., *Aacs*, *Acaca*, and *Fasn*; Figure 4C) targeted by the all three upstream regulators. These genes are, as mentioned above, responsible for lipogenesis. The regulatory roles of *Srebf1* in lipid metabolism, specifically de novo lipogenesis, have been highlighted elsewhere [25,26,27,28]. In our study, a decrease in *Srebf1* was predicted (Figure 4C) and was validated by the qPCR analysis in IDH2 KO mice liver tissues (Figure 7A). This supports well established roles of *Srebf1* that control the expression of lipogenic genes such as *Thrsp*, *Acaca,* and *Me1* [29,30,31]. In particular, *Srebf1* enhances the *Acaca* expression by forming a tetrameric complex with TR/RXR [32], which is important to note because *Acaca* is an earlier target of *Srebf1* in the lipogenesis process, followed by *Fasn*, *Scd1*, and other lipogenic genes. Similarly, the dramatic down-regulation of *Srebf1* and SREBP1 (i.e., encoded by *Srebf1*) in liver tissues of IDH2 KO mice was reported [24].

The *Srebf1*-mediated transcriptional activation of de novo lipogenesis is well-established and there are auxiliary genes supporting lipogenesis indirectly by providing NADPH [26]. The expressions of these auxiliary genes are also regulated by *Srebf1*. For instance, the over-expression of hepatic *Srebf1* upregulates the transcription of lipogenic genes, which are involved in NADPH production [28,33]. This might suggest there is an indirect regulatory role of IDH2 in lipogenesis because *Idh2* is also one of the major NADPH producing enzymes. Further, *Idh2* gene transcription might be regulated in a sterol dependent manner in hepatocytes [34]. In this study, *Idh1* transcription was activated by both *Srebf1* and *Srebf2.* Although *Idh1* showed a stronger association with sterols (2.3-fold induction of *Idh1* mRNA by sterol depletion), a regulatory role of sterols in *Idh2* expression was clearly observed (1.3-fold induction of *Idh2* mRNA by sterol depletion). In this study, the IDH2 deficiency led the downregulation of both *Srebf1* and *Srebf2* in the qPCR or transcriptomics analyses, suggesting that there might be a feedback mechanism between these genes. Taken together, the previous and current studies represent a clear association between *Srebfs* and *Idh*. However, further investigation is warranted to unravel the causal relationship of *Srebfs* expression in IDH2 KO mice.

In addition to the IPA database, DAVID tools were utilized, which annotated that the lipid metabolic process is the major process influenced in IDH2 KO mice (Figure 5). Specifically, the AMPK signaling pathway was predicted as the most enriched pathway with DEGs (Figure 6). AMPK, known to be activated in response to an increased AMP/ATP ratio or ADP/ATP ratio [35,36], is a master regulator of both lipogenesis and lipolysis. Of note, activated AMPK inhibits *Srebf1*, followed by decreasing downstream lipogenic genes [37]. This prediction is plausible since *Idh2* deletion causes ATP depletion in several tissues such as the heart, white adipose tissue, and kidney [7,8,38]. Interestingly, the AMPK-mediated lipid metabolism can also be directly modulated by thyroid hormones [39,40]. The thyroid hormone analogues act differently on lipid metabolism. For instance, T2 thyroid hormone is known to exert anti-lipogenic activity against high-fat diet intervention through the direct activation of *Sirt1* [41]. On the other hand, T3 thyroid hormone up-regulates *Acaca* expression by binding to *Acaca* gene promoters [32,42,43]. Therefore, our results indicate that there might be possible links between *Idh2* and thyroid hormone homeostasis in relation to the AMPK pathway. However, this is beyond the scope of the study but clearly requires further investigations.

The accumulating evidences indicate that the excessive production of mitochondrial ROS (mtROS) damages cellular components as well as induces cytotoxicity and senescence [44]. In that regard, *Idh2* deficient mice have been utilized as a model of mitochondrial-related diseases since IDH2 is one of the major NADPH generating enzymes in mitochondria [45]. In line with this, multiple studies, including our previous works, demonstrated that *Idh2* deletion significantly increases mtROS production, thereby increasing susceptibility to diseases [9,24,38]. Previously, the authors reported that the mtROS level was elevated, while lipogenesis was suppressed in *Idh2* deficient mice [8,24]. These are similar results to the current findings (although mtROS level was not measured in this study). Thus, it was speculated that the suppression of lipogenesis might be somehow related with elevated thermogenesis, possibly via *Ucp1* [8]. This is strengthened by a recent finding showing that mtROS might be a non-canonical activator of AMPK [46]. Additionally, another recent study reported that mtROS-mediated AMPK activation suppresses lipogenesis in lung adenocarcinoma cell lines [47]. In fact, the AMPK signaling pathway was predicted as the most enriched pathway in the present study (Figure 6). Therefore, it is possible to hypothesize that *Idh2* deletion, due to the accumulated mtROS, activates the AMPK, thus upregulates the genes involved in β-oxidation (e.g., *Ppargc1*, *Ucp1*), which leads to the suppression of lipogenesis. Further mechanistic studies are warranted to elucidate if mtROS accumulation is a precedent event for lipogenesis suppression in *Idh2* deficient mice.

In summary, the current study is the first attempt to investigate comprehensive transcriptome signatures in liver tissues of IDH2 KO mice compared to WT mice. The online knowledge-based software analyses and qPCR validation indicated that IDH2 might be primarily responsible for the regulation of hepatic lipid metabolism (more specifically, de novo lipogenesis suppression). A combined analysis of the IPA upstream analysis and KEGG pathway analysis predicted that AMPK activation, likely resulting from an increased AMP/ATP ratio in IDH2 KO mice, inhibits lipogenic genes via *Srebf1*. It was also suggested that the thyroid hormone may play a key role in the regulation of lipogenesis in IDH2 KO mice via the TR/RXR activation. Since our unbiased study, for the first time, provided evidence on transcriptome characteristics of IDH2 KO mice liver, further investigations are warranted. For example, the implications of thyroid hormones on lipid metabolism in IDH2 KO mice are likely to provide deeper insights. Moreover, based on our report, the physiological roles of *Idh2* can be reappraised in a different perspective rather than controlling ROS homeostasis.

## Figures and Tables

**Table 1:** Nine networks of differentially expressed genes ^1^ and their biofunctions predicted by Ingenuity Pathway Analysis (IPA) software.

Rank	Molecules in Network	Score ^2^	Focused Molecule ^3^	Top Functional Networks
1	AHCY, ALAS1, AMFR, Apoc3, C1S, C3, CAT, CEBPD, Ces2a, Clec2d, CLOCK, CXCL3, Cyp2c40, CYP2C9, CYP2E1, Cyp4a14, DHCR7, DNAJC7, EEF2K, FOS, G0S2, Gstm6, HES1, HMGCL, HSF2, ICAM1, IL1B, NFE2L1, NR1I2, PPARA, PPARGC1A, RXRA, ST3GAL5, SULT2A1, TXNIP	21	17	Lipid Metabolism, Molecular Transport, Small Molecule Biochemistry
2	AACS, ACACA, ACACB, AGPAT2, BTC, CCND3, CEBPA, Cyp2c12, DUSP1, ELOVL2, FABP5, FASN, FOS, HSD3B7, KLB, MBTPS1, MID1IP1, MLX, MLXIPL, NCOR1, NEUROG3, NFIL3, ONECUT1, PRKAA2, PSME3, RARG, RXRA, SREBF2, SRSF2, SULT2A1, THRSP, TKFC, TRIB1, Ugt1a7c, VDR	19	16	Lipid Metabolism, Molecular Transport, Small Molecule Biochemistry
3	ARNT, C3, Calm1, CEBPD, CIAPIN1, DDC, DNM2, EGF, EGR1, ESR1, FOS, GDF15, HAMP, HDAC3, ICAM1, IL6ST, JAK2, MAPK3, ME1, MTOR, NCOA1, PCNA, PDE4A, PPP2CB, PRKAR1A, RGS16, RGS3, RICTOR, RPS15, RPS4Y1, RPS6KB1, SDHB, SERPINA1, SP1, STAT3	19	16	Cell Death and Survival, Cancer, Hematological Disease
4	DYNC1H1, S100A10	1	1	Cellular Growth and Proliferation, Developmental Disorder, Hereditary Disorder
5	DLG1, GJB1	1	1	Carbohydrate Metabolism, Cell-To-Cell Signaling and Interaction, Cellular Function and Maintenance
6	GATA1, MYCN	1	1	Cancer, Hematological Disease, Organismal Injury and Abnormalities
7	EIF2AK2, TUBA1A	1	1	Developmental Disorder, Hereditary Disorder, Neurological Disease
8	ADAM15, MBD2	1	1	Cell-To-Cell Signaling and Interaction, Hair and Skin Development and Function, Cellular Compromise
9	PRKAA1, PRKAB1, PRKAG1	1	1	Protein Synthesis, Cell Morphology, Cellular Function and Maintenance

^1^ Differentially expressed genes were subjected to the IPA software to identify highly-interconnected networks and enriched genes representing significant biological function in mice liver transcriptome influenced by deletion of IDH2. ^2^ Scores were calculated for each Functional Network based on relevance of the network to the focused molecules on the IPA software. This indicates a significance of link between Molecules in Networks and Top Functional Networks based on the number of focused molecules and the size of the network to approximate the relevance of the network to the original list of focused molecules. ^3^ Genes present in our differentially expressed genes.

## Supplementary Materials

The following are available online at https://www.mdpi.com/2073-4425/10/9/728/s1, Table S1: A list of differentially expressed genes in RNA sequencing dataset, Table S2: DAVID GO terms for biological process, Table S3: Annotation clusters of DAVID GO terms for biological process, Table S4: A list of representative terms in Kyoto Encyclopedia of Gene and Genomes (KEGG) pathways, Table S5: A list of TaqMan primers.
