# Supplementary material for: Hepatic Transcriptomics Reveals that Lipogenesis Is a Key Signaling Pathway in Isocitrate Dehydrogenase 2 Deficient Mice
Source: Genes (Basel). 2019 Sep 19;10(9):728. doi: 10.3390/genes10090728 (PMC6770969; doi:10.3390/genes10090728)
Supplement: Supplementary file 1 [file genes-10-00728-s001.pdf]

Table S1. A list of differentially expressed genes[1] in RNA sequencing dataset

Gene symbol	Fold change	P-value
Tmc7	-8.696	0.00766
Rgs3	-6.826	0.00312
Gopc	-5.313	0.00214
Cnbd2	-5.176	0.01026
Eef2k	-4.981	0.00250
Fabp5	-4.881	0.03206
Dnm2	-4.564	0.00250
Me1	-4.563	0.01045
St3gal5	-4.538	0.00298
Man2b1	-4.125	0.01554
Dctn4	-4.094	0.03106
Hmgcl	-4.062	0.01787
Amfr	-4.043	0.00515
Ddc	-3.896	0.01715
Prkag1	-3.890	0.00390
Tmem245	-3.854	0.03777
Serpina1a	-3.808	0.03518
Aspg	-3.737	0.03287
Serpina3a	-3.715	0.01926
Onecut1	-3.693	0.04203
TrnS2	-3.615	0.03711
Gstm6	-3.610	0.02624
Ppp2cb	-3.597	0.01545
Ugt1a7c	-3.553	0.01225
Aacs	-3.547	0.01346
Gpatch8	-3.543	0.02193
Bag2	-3.474	0.00053
Rsph1	-3.450	0.02809
Orai1	-3.444	0.01262
Rtn3	-3.443	0.00188
Tmem53	-3.390	0.03934
Clec2d	-3.382	0.04926
Vps53	-3.366	0.00210
S100a10	-3.292	0.00529
Slc50a1	-3.234	0.02470
Ihh	-3.203	0.04394
Rxra	-3.199	0.01079
Elovl2	-3.167	0.02657

Rps4x	-3.152	0.00687
Srebf2	-3.064	0.00399
Cdkn2aipnl	-3.052	0.03147
Stam	-2.977	0.01036
Wdr5b	-2.968	0.04049
Cebpa	-2.938	0.03513
Mob4	-2.883	0.02727
Cyp2c68	-2.880	0.03067
Acaca	-2.855	0.01196
Usp39	-2.838	0.00818
Tbc1d32	-2.832	0.04589
Blvra	-2.822	0.03946
Snrpf	-2.751	0.01925
Btaf1	-2.736	0.03662
Tkfc	-2.727	0.00417
Plpp5	-2.727	0.03715
Nudcd1	-2.704	0.04575
Yipf3	-2.670	0.02526
Fdx1l	-2.669	0.00698
Calm1	-2.659	0.03733
Fasn	-2.652	0.03175
Zfp180	-2.623	0.04431
Sdhb	-2.563	0.04137
C1s2	-2.537	0.03181
Arhgef11	-2.533	0.03831
Arhgap15os	-2.497	0.04012
Srgap3	-2.469	0.02968
Ryk	-2.462	0.04867
Prkar1a	-2.450	0.01945
Tm6sf2	-2.412	0.03439
Sf3b2	-2.400	0.04698
Urah	-2.373	0.00939
Scd3	-2.310	0.01819
Trim34a	-2.275	0.01030
Thrsp	-2.237	0.04215
Rnf181	-2.233	0.01102
Mtg1	-2.226	0.04726
Mpc1	-2.193	0.01321
Dusp22	-2.188	0.04920
Dnajc7	-2.186	0.02859
Wdr70	-2.163	0.01798

Swi5	-2.159	0.02130
Copz1	-2.157	0.02307
Rrbp1	-2.150	0.04093
Wdr6	-2.144	0.03727
Rps15	-2.144	0.01192
Hsd3b7	-2.144	0.01456
Pmvk	-2.107	0.03961
Tma16	-2.083	0.04844
Mycn	-2.080	0.02839
Slc25a11	-2.063	0.00480
Ces2a	-2.057	0.00189
Gja4	2.009	0.00270
Mup-ps16	2.011	0.04858
Tia1	2.014	0.04227
Enc1	2.020	0.04158
Dgcr14	2.041	0.03075
Inafm1	2.056	0.04793
Cngb1	2.111	0.02270
Scamp3	2.123	0.02600
Ngrn	2.129	0.01282
Zfp608	2.150	0.02774
Mansc4	2.157	0.00786
Pde4a	2.160	0.00634
Rbm3	2.162	0.03389
Gpr137c	2.162	0.01115
Ccnh	2.177	0.01316
Pdk3	2.205	0.02368
Spag1	2.218	0.00483
Uimc1	2.221	0.00553
Nle1	2.312	0.02224
Ciapin1	2.313	0.00004
Hsf2	2.322	0.03259
Simc1	2.332	0.01828
Ptprcap	2.340	0.01870
Ppp1r3fos	2.348	0.04604
Osbpl5	2.352	0.04332
Pcp4l1	2.379	0.04499
Ccdc73	2.396	0.04686
Mustn1	2.401	0.03134
Fam104a	2.420	0.04288
Rtn1	2.421	0.01884

Tmem194b	2.467	0.02851
Mkln1os	2.535	0.04120
Neurl4	2.555	0.00997
G0s2	2.587	0.02171
Slamf8	2.645	0.02847
Fstl4	2.670	0.03646
Atad3aos	2.741	0.02023
Mocs1	2.840	0.02320
Tuba1a	2.870	0.00568
Rrnad1	2.895	0.02550
Fam83f	3.043	0.00100
Hes1	3.045	0.01572
Micalcl	3.086	0.00819
Dlg4	3.097	0.00512
Adat3	3.113	0.01602
Trbc2	3.142	0.04632
Apold1	3.166	0.02553
Ppp1r1c	3.208	0.00553
Lpcat2	3.224	0.03083
Igkc	3.390	0.02047
Aard	3.470	0.01320
Gjc3	3.511	0.00132
Cirbp	3.545	0.02555
Trim34b	3.629	0.01340
Krt23	3.670	0.01669
Ccdc159	3.745	0.00855
Dusp1	3.809	0.02923
Zfp36	3.849	0.02651
Cyp4a14	3.926	0.01863
Dlg1	4.203	0.01470
Fbxo44	4.267	0.01264
Hba-a2	4.298	0.02169
Plscr2	4.481	0.00334
Junb	4.602	0.00091
Ier2	4.620	0.01199
Gdf15	4.716	0.03223
Hba-a1	5.127	0.01746
Adam15	5.165	0.00623
Hddc3	5.299	0.00050
Igha	6.433	0.00767
Hbb-bs	6.530	0.00815

Ighv1-81	9.549	0.03563
Fos	10.774	0.04110
Egr1	16.496	0.00299
Mettl7a2	23.132	0.01850
Sult2a3	31.485	0.02596
Gdpd3	81.848	0.02006

[1] Genes passed following criteria were selected as differentially expressed genes: Fold change > ±2, and P-value < 0.05

Table S2. DAVID GO terms for biological process

Category[1]	Terms[2]	Count[3]	%	P-Value
GOTERM_BP_FAT	Lipid Metabolic Process	21	12.7	5.30E-04
GOTERM_BP_FAT	Organonitrogen Compound Biosynthetic Process	21	12.7	1.70E-03
GOTERM_BP_FAT	Amide Biosynthetic Process	16	9.6	8.20E-04
GOTERM_BP_FAT	Organic Acid Metabolic Process	16	9.6	1.90E-03
GOTERM_BP_FAT	Response to Organic Cyclic Compound	16	9.6	6.70E-03
GOTERM_BP_FAT	Cellular Amide Metabolic Process	16	9.6	8.60E-03
GOTERM_BP_FAT	Carboxylic Acid Metabolic Process	15	9.0	2.00E-03
GOTERM_BP_FAT	Oxoacid Metabolic Process	15	9.0	2.10E-03
GOTERM_BP_FAT	Hemopoiesis	15	9.0	5.70E-03
GOTERM_BP_FAT	Cellular Lipid Metabolic Process	15	9.0	7.10E-03
GOTERM_BP_FAT	Hematopoietic or Lymphoid Organ Development	15	9.0	8.90E-03
GOTERM_BP_FAT	Translation	14	8.4	2.20E-03
GOTERM_BP_FAT	Peptide Biosynthetic Process	14	8.4	2.90E-03
GOTERM_BP_FAT	Cofactor Metabolic Process	11	6.6	4.20E-04
GOTERM_BP_FAT	Lipid Biosynthetic Process	11	6.6	6.80E-03
GOTERM_BP_FAT	Monocarboxylic Acid Metabolic Process	11	6.6	7.50E-03

[1] Functional Annotation Tool (FAT) GO category inside a cluster

[2] GO terms inside each cluster

[3] Gene count in differentially expressed genes

Table S3. Annotation clusters of DAVID GO terms for biological process

Annotation Cluster 1	Enrichment Score[1]: 3.2	Count[2]	P-Value
GOTERM_BP_FAT	Acyl-CoA Metabolic Process	6	3.20E-04
GOTERM_BP_FAT	Thioester Metabolic Process	6	3.20E-04
GOTERM_BP_FAT	Cofactor Metabolic Process	11	4.20E-04
GOTERM_BP_FAT	Acetyl-CoA Metabolic Process	4	1.20E-03
GOTERM_BP_FAT	Coenzyme Metabolic Process	9	1.90E-03
Annotation Cluster 2	Enrichment Score: 2.53	Count	P-Value
GOTERM_BP_FAT	Lipid Metabolic Process	21	5.30E-04
GOTERM_BP_FAT	Lipid Biosynthetic Process	11	6.80E-03
GOTERM_BP_FAT	Cellular Lipid Metabolic Process	15	7.10E-03
Annotation Cluster 3	Enrichment Score: 2.49	Count	P-Value
GOTERM_BP_FAT	Amide Biosynthetic Process	16	8.20E-04
GOTERM_BP_FAT	Organonitrogen Compound Biosynthetic Process	21	1.70E-03
GOTERM_BP_FAT	Translation	14	2.20E-03
GOTERM_BP_FAT	Peptide Biosynthetic Process	14	2.90E-03
GOTERM_BP_FAT	Regulation of Translation	9	5.80E-03
GOTERM_BP_FAT	Cellular Amide Metabolic Process	16	8.60E-03
GOTERM_BP_FAT	Regulation of Cellular Amide Metabolic Process	9	8.70E-03
Annotation Cluster 4	Enrichment Score: 2.48	Count	P-Value
GOTERM_BP_FAT	Organic Acid Metabolic Process	16	1.90E-03
GOTERM_BP_FAT	Carboxylic Acid Metabolic Process	15	2.00E-03

GOTERM_BP_FAT	Oxoacid Metabolic Process	15	2.10E-03
GOTERM_BP_FAT	Cellular Lipid Metabolic Process	15	7.10E-03
GOTERM_BP_FAT	Monocarboxylic Acid Metabolic Process	11	7.50E-03
Annotation Cluster 5	Enrichment Score: 2.14	Count	P-Value
GOTERM_BP_FAT	Hemopoiesis	15	5.70E-03
GOTERM_BP_FAT	Myeloid Cell Differentiation	9	7.50E-03
GOTERM_BP_FAT	Hematopoietic or Lymphoid Organ Development	15	8.90E-03

[1] Minimum enrichment score threshold was set to 0.75.

[2] Gene count in differentially expressed genes

Table S4. A list of representative terms in Kyoto Encyclopedia of Gene and Genomes (KEGG) pathways

Category	Term	Count	P-value
KEGG_PATHWAY	AMPK Signaling Pathway	6	0.005
KEGG_PATHWAY	Fatty Acid Metabolism	4	0.010
KEGG_PATHWAY	PPAR Signaling Pathway	4	0.034
KEGG_PATHWAY	Insulin Signaling Pathway	5	0.035
KEGG_PATHWAY	African Trypanosomiasis	3	0.038
KEGG_PATHWAY	Chemical Carcinogenesis	4	0.048
KEGG_PATHWAY	Malaria	3	0.067
KEGG_PATHWAY	cAMP Signaling Pathway	5	0.096

Table S5. A list of TaqMan primers

Primer	Manufacturer	Assay ID	RefSeq/ Accession number
β-Actin (*Actb*)	Life Technologies	Mm02619580_g1	NM_007393
Acetyl CoA carboxylase alpha (*Acaca*)		Mm01304257_m1	NM_133360.2
Fatty acid synthase (*Fasn*)		Mm00662319_m1	NM_007988.3
Stearoyl-CoA Desaturase-1 (*Scd1*)		Mm00772290_m1	NM_009127.4
Retinoid X receptor alpha (*Rxra*)		Mm00441185_m1	NM_001290481.1
Thyroid hormone responsive (*Thrsp*)		Mm01273967_m1	NM_009381.2
Malic enzyme (*Me1*)		Mm00782380_s1	NM_001198933.1
CD36 antigen (*Cd36*)		Mm00432403_m1	NM_001159555.1
Solute carrier family 27 member 1 (*Slc27a1*)		Mm00449511_m1	NM_011977.3
Sterol regulatory element binding transcription factor 1 (*Srebf1*)		Mm00550338_m1	NM_011480.3
Peroxisome proliferator activated receptor alpha (*Ppara*)		Mm00440939_m1	NM_001113418.1
Sirtuin 1 (*Sirt1*)		Mm01168521_m1	NM_001159589.1
Peroxisome proliferative activated receptor, gamma, coactivator 1 alpha (*Ppargc1*)		Mm01208835_m1	NM_008904.2
ELOVL family member 6 (*Elovl6*)		Mm00851223_s1	NM_130450.2
Diacylglycerol O-acyltransferase 2 (*Dgat2*)		Mm00499536_m1	NM_026384.3
Acyl-CoA oxidase 1, palmitoyl (*Acox1*)		Mm01246834_m1	NM_001271898.1
